# Knotify: An Efficient Parallel Platform for RNA Pseudoknot Prediction Using Syntactic Pattern Recognition

**DOI:** 10.3390/mps5010014

**Published:** 2022-02-02

**Authors:** Christos Andrikos, Evangelos Makris, Angelos Kolaitis, Georgios Rassias, Christos Pavlatos, Panayiotis Tsanakas

**Affiliations:** 1 School of Electrical and Computer Engineering, National Technical University of Athens, 9 Iroon Polytechniou St., 15780 Athens, Greece; candrikos@cslab.ece.ntua.gr (C.A.); vmakris@mail.ntua.gr (E.M.); akolaitis@mail.ntua.gr (A.K.); grassias@cslab.ece.ntua.gr (G.R.); panag@cs.ntua.gr (P.T.); 2 Hellenic Air Force Academy, Dekelia Air Base, Acharnes, 13671 Athens, Greece

**Keywords:** RNA secondary structure, pseudoknot, syntactic pattern recognition, context-free grammar

## Abstract

Obtaining valuable clues for noncoding RNA (ribonucleic acid) subsequences remains a significant challenge, acknowledging that most of the human genome transcribes into noncoding RNA parts related to unknown biological operations. Capturing these clues relies on accurate “base pairing” prediction, also known as “RNA secondary structure prediction”. As COVID-19 is considered a severe global threat, the single-stranded SARS-CoV-2 virus reveals the importance of establishing an efficient RNA analysis toolkit. This work aimed to contribute to that by introducing a novel system committed to predicting RNA secondary structure patterns (i.e., RNA’s pseudoknots) that leverage syntactic pattern-recognition strategies. Having focused on the pseudoknot predictions, we formalized the secondary structure prediction of the RNA to be primarily a parsing and, secondly, an optimization problem. The proposed methodology addresses the problem of predicting pseudoknots of the first order (H-type). We introduce a context-free grammar (CFG) that affords enough expression power to recognize potential pseudoknot pattern. In addition, an alternative methodology of detecting possible pseudoknots is also implemented as well, using a brute-force algorithm. Any input sequence may highlight multiple potential folding patterns requiring a strict methodology to determine the single biologically realistic one. We conscripted a novel heuristic over the widely accepted notion of free-energy minimization to tackle such ambiguity in a performant way by utilizing each pattern’s context to unveil the most prominent pseudoknot pattern. The overall process features polynomial-time complexity, while its parallel implementation enhances the end performance, as proportional to the deployed hardware. The proposed methodology does succeed in predicting the core stems of any RNA pseudoknot of the test dataset by performing a 76.4% recall ratio. The methodology achieved a F1-score equal to 0.774 and MCC equal 0.543 in discovering all the stems of an RNA sequence, outperforming the particular task. Measurements were taken using a dataset of 262 RNA sequences establishing a performance speed of 1.31, 3.45, and 7.76 compared to three well-known platforms. The implementation source code is publicly available under knotify github repo.

## 1. Introduction

The RNA molecule, being the intermediate representation of the information flowing from DNA to proteins, holds a crucial role in many biological processes. Recent studies reveal that its detailed structural analysis is of utmost importance. The RNA structure is often depicted by a 2-D representation of the base-pairing nucleotide (A-U, C-G, and G-U pairs) known as the “secondary structure”, which conduces to the more-complex construction 3-D representation, termed the tertiary structure. The substantial role of RNA in the expression of proteins, along with its contribution to the gene expression regulation, catalysis, and site recognition, requires the enlightenment of its territorial structure, which is associated with considerable biological functionalities.

More specifically, non-coding RNAs (ncRNAs) are functional RNA molecules transcripted from DNA but not translated into proteins. The latter must not be misinterpreted as not enclosing important information or contributing to any biological operation. Current evidence implies that ncRNAs transcribe most of the genomes of mammals and other complex bions, to contradict the widespread assumption that proteins transcribe most genetic information. Their purpose is to fulfill diverse catalytic and structural functions, along with regulating gene expressions at the transcriptional and post-transcriptional level.

The literature contains a considerable number of publications discussing the prediction of RNA secondary structure. The employed methodologies are mainly based on dynamic programming algorithms, thermodynamic models, stochastic methods, and syntactic pattern-recognition techniques. A thorough analysis of the related work is presented in Section 3. In this study, a methodology of predicting H-type RNA pseudoknots, a familiar yet complex structure of the RNA secondary representation, was granted. Initially, the RNA raw string was parsed via a context-free grammar parser for all trees that include a pseudoknot to be produced. Next, all trees are traversed to identify additional base pairs around the pseudoknot. Finally, the optimal tree was selected, maximizing the number of base pairs, while minimizing the free energy of the pseudoknot. For the first task, i.e., detecting possible pseudoknots, an alternative methodology was implemented as well using a brute force algorithm. The proposed methodology succeeded in predicting the core stems of any RNA pseudoknot of the test dataset by performing a 76.4% recall ratio. It achieved a F1-score equal to 0.774 and a MCC equal 0.543. A dataset [1] of 262 RNA sequences was used, proving a performance speed of 1.31, 3.45, and 7.75 compared to three well-known platforms [2,3].

The article is organized at the following sections. In Section 2, all required definitions and preliminaries are given. Section 3 presents any related publications thoroughly. In Section 4, the proposed methodology is analyzed, and an illustrative example is presented. Section 5 focuses on implementation details, while, experimental results for several RNA structures are discussed in Section 6. In the end, Section 7 concludes the presented methodology and implementation and describes future modifications and improvements.

## 2. Theoretical Background

Non-coding RNAs (ncRNAs) are functional RNA molecules that are transcripted from DNA but not translated into proteins. This is not to say that ncRNAs do not provide knowledge or serve a purpose. Although it has been generally assumed that proteins transcribe most genetic information, recent evidence suggests that ncRNAs transcribe most of the genomes of mammals and other complex bions. Their purpose is to fulfill diverse catalytic and structural functions and regulate gene expressions at the transcriptional and post-transcriptional level.

### 2.1. RNA

RNA is the cornerstone of various biological processes; it carries genetic information that is encoded into four distinct nitrogenous bases, i.e., adenine (A), cytosine (C), guanine (G), and uracil (U). As a messenger [4], it acts as a regulator for gene expression [5] or even as a catalyst [6] to complex biological operations. Recent studies reveal its contribution to functions not directly related to protein conformation [7,8].

Contrary to DNA, RNA features a single-stranded molecule resulting in a solid inclination of its bases to interact in two principal ways, either perpendicular to their planes (stacking) or hydrogen-bonded within the base planes (pairing). Those interactions form base pairs via hydrogen bonds between the corresponding nucleotide, i.e., the standard set of RNA base-pairs (AU, GC) known as Watson–Crick base-pairs [9] and the regularly appearing GU wobble-base pairs. On top of that, RNA bases also form bonds between their Hoogsteen- or CH-edge and their sugar-edge to allow “pairing” between three bases at once, known as base-triplets. In this setting, RNA molecules usually come as single strands, but they tend to fold themselves into what is known as their tertiary structure, which determines the spatial stability of its single-stranded helix. Simple linear strings of RNA form various complex three-dimensional structures due to the exact hydrogen-bonding mechanism stabilizing the well-known DNA double helix. Helices, also known as stems, are formed in the case of RNA in an intra-molecular way.

According to the literature, there is a strong correlation between the structure of a RNA molecule and its function [10,11,12] to the extent of determination. Accurate RNA secondary-structure predictions highlight the RNA’s functionality. For instance, according to work in [13], RNA secondary structure determines vital responsibility in the central nervous system that may be relevant to the aetiology of neurological disorders. For quite some time, researchers have been working on identifying and accurately measuring the structural elements of RNA to monitor their structure. To identify the RNA structure, chemical mapping has been proposed in works such as [14]; however, these methods are not generalizable and often have biases in their reactivities. A compelling method widely used for measuring RNA structure in vitro is SHAPE [15] and its transformation icSHAPE [16] for in vivo measuring. Other methods such as X-ray crystallography [17] and nuclear magnetic resonance (NMR) are time-consuming and cost-inefficient. For all the experimental methods, the accuracy is restricted by the length of the RNA, i.e., the prediction accuracies are higher for shorter RNAs. Therefore, in RNAs of more considerable lengths, secondary-structure prediction provides a significant key to deciphering their potential functions.

#### The Pseudoknot Pattern

**Figure 1:**
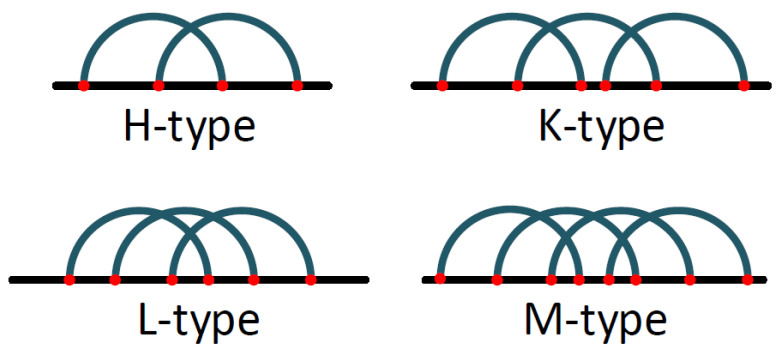
Four basic types of pseudoknots.

The pseudoknot pattern is one of the most-common RNA-folding motifs. It was first identified in the *Turnip Yellow Mosaic virus* [18], consisting of two helical segments that are bound by at least two single-stranded sections or loops. Pseudoknots are met into various folding motifs. In general, there are four basic types [19] of pseudoknots that have been distinguished (i) the H-type, (ii) the K-type, (iii) the L-type, and (iv) the M-type as shown in Figure 1.

The most prevalent is the H-type [20], which is the one this work mainly focused on. In this pseudoknot pattern, the single-stranded RNA sequence folds into an “S” fashion to form two tangent loops, each one of arbitrary length. The two base pairs can be piled on top of each other to develop an essentially continuous helix. During the pseudoknot folding formation, the single-stranded loop parts consist instantaneously of bases, which urge to form hydrogen bonds with any adjacent “free” neighbor to contribute to the overall arrangement of the RNA molecule.

Even though a pseudoknot is a typical pattern, it is the springboard for remarkable yet robust RNA structures. Being a structurally diverse group varying in length and loops, and stems (the hydrogen-bonded base-pairs), pseudoknots are related with a plethora of biological operations such as holding the catalytic role of various ribozymes [21,22], self-splicing introns [23], and telomerase [24]. Pseudoknots are even contributing, sometimes to the extent of definition, to the alternation of the gene expression of many viruses [25,26,27].

### 2.2. Syntactic Pattern Recognition

The underlying model of the proposed predicting methodology of pseudoknots in RNA structures is that of syntactic pattern recognition. In syntactic pattern recognition, a language is defined as a set of syntax rules, which may construct a string belonging to that language [28]. The set of syntax rules is part of a grammar and determines the way accurate strings of symbols, which are components of the defined language, may be produced [28]. All grammars belong into four specific classes defined by Noam Chomsky [29], which is acknowledged as the Chomsky hierarchy. Context-free grammars (CFG) are one of those four categories and are widely used for the implementation of programming languages and human-language recognition [30].

#### 2.2.1. Context Free Grammars

The formal definition of a CFG [31] is a group of four sets (quadruple), i.e., CFG=〈NT,T,R,S〉. *S* (S∈NT) is the start symbol, which is also called the root of the grammar. *T* is a set that includes all terminal symbols, while NT includes all non-terminal symbols. All syntactic rules are contained in set *R*. The syntax rules follow the formalism C→δ, where C∈NT and $\delta \in {\left(T\cup NT\right)}^{*}$, defining that symbol *C*, which is a non-terminal symbol, may be altered by δ.

Latin capital characters are used to represent the non-terminal symbols, while Latin lowercase characters are used to represent terminal symbols. Greek lowercase characters represent strings of terminals and non-terminal symbols.

Parsing is the process that makes use of the syntax rules of a grammar, in order to produce a string, meaning it validates a string’s syntactic exactitude. When a methodology solely resolves if a string of symbols may be produced by a grammar, then it forms a recognizer. In case a methodology, apart from recognizing a string, constructs a parse tree as well, then it is called parser. The root of a parse tree is the root symbol of the grammar; each node of the parse tree is formed by a syntax rule; and the leaves of the parse tree are terminal symbols forming the recognized string. A grammar is called ambiguous when a string can have more than one leftmost derivation or parse tree.

#### 2.2.2. Primitive Pattern Selection

During the design process of an implementation based on syntactic pattern recognition, it is really vital to select the appropriate primitive patterns. In case of a RNA sequence that consists of the four key bases of adenine, guanine, cytosine, and uracil, the most-common case is to consider RNA as a string of symbols *a*, *g*, *c*, and *u* such as *auacggc* or *cugcaucccgcauauacg*. Consequently, the vocabulary of a grammar aiming to recognize strings representing RNA should contain only four terminal symbols T = (*a*, *g*, *c*, and *u*).

#### 2.2.3. CFG Parsers

Due to the high expressive capability of CFG grammars, numerous parsing algorithms have been proposed for them. Two well-known CFG parsing algorithms are those proposed by Cocke, Younger, and Kasami (CYK) [32] and by Earley [33]. Based on the two algorithms mentioned above, several worth-mentioning extensions [34,35,36] and parallel versions [37,38] exist in the literature as well.

Earley and CYK are algorithms of comparable complexity [34], as both of them have adopted a similar dynamic programming method. Earley’s parser was selected for the proposed implementation due to his efficiency and ability to handle ambiguous grammars.

#### 2.2.4. Earley’s Parsing Algorithm

The parsing algorithm for CFG grammars presented by Earley in 1970 constructs the parse tree using a top-down methodology. Earley’s algorithm locates the dot symbol “${}_{•}$” ∉(N∪NT), in each rule producing dotted rules. The existence of a dot in a rule indicates that the part of the rule left of the dot has been recognized, while the part of the rule right of the dot has not yet been recognized. In case a dot reaches the last position of a rule that has the root symbol at its left side, then the input string is considered recognized. This algorithm defines and applies operations, which are named Scanner, Predictor, and Completer. The input string α=a${}_{1}$a${}_{2}$a${}_{3}\ldots$a${}_{n}$ is traversed from a${}_{1}$ to a${}_{n}$. As each input symbol is scanned, a data set is constructed, representing the state of the recognition procedure at this place in the scan. Consequently, the algorithm builds n+1 data sets of states. A state is simply a set of three integers {SR,p,F}. SR indicates the number of the rule; *p* is the position of symbol “${}_{•}$”; and *F* is the enumeration of the set where the dotted rule was initially generated. A state in an Earley’s data set is of form i:${}_{F}Y\to \alpha_{•}Z\gamma$, meaning syntactic rule Y→αZγ having symbol “${}_{•}$” at the *p*th position (|α|=p), initially generated at the *F*th data set and is located in data set $S_{i}$. As the reading of the input symbols is moving on, new data sets of dotted rules are generated. The three operations are sequentially applied to each dotted rule of all sets. The presence of a completed dotted rule having a root symbol at the left side of the rule in the last data set denotes the recognition of the input string.

The implementation of the proposed method is based on a Yet Another Early Parser (YAEP) parser [39], which is one of the most efficient Early’s parser implementations capable of parsing ambiguous grammars as well.

The Earley’s parser algorithm is presented in Algorithm 1. In the main function EARLEY_PARSER, an array of sets containing states is initialized according to input sting length (INITIALIZE(input_string)) and adds a state having “${}_{•}$” to the left side of start symbol S at the set with enumeration 0 ( ADD_TO_SET((Start →${}_{•}$ S, 0), Sets[0])). Then, a double-nested loop is executed. The nested loop examines each state in each set, and a set may expand during this loop as the three operations are adding states to sets. In each state, it is examined whether the right of “${}_{•}$” is a nonterminal symbol, a terminal symbol, or the state is completed (“${}_{•}$” is at the end of the rule). and the functions are called PREDICTOR, SCANNER, or COMPLETER, respectively.

In case function PREDICTOR is called, then for the nonterminal symbol that is right of “${}_{•}$” (nonterminal symbol C in pseudocode as dotted rule is B →α${}_{•}$ C β), all grammar rules are traversed to select the rules that have this symbol at the left side of the rule (C →δ). The selected rules are then added to this set after placing the “${}_{•}$” at the first position of the right side of the rule (C →${}_{•}$δ).

When function SCANNER is called, if the terminal symbol that is right of “${}_{•}$” (terminal symbol a in pseudocode as dotted rule is B →γ${}_{•}$ a δ) is equal to the current examined symbol of the input sting (input_string[i]), this state is added to the next set after moving “${}_{•}$” one position to the right (B →γ a ${}_{•}$δ).

When function COMPLETER is called, the states in the set where the completed state (A →δ${}_{•}$) was initially generated (x in pseudocode) are traversed in order to select the states that have the symbol (A in pseudocode) at the left side of the rule, one position after the “${}_{•}$” (B →γ${}_{•}$Aβ). These states are added in Sets[i] after moving the dot one position to the right (B →γ A ${}_{•}$β).
**Algorithm 1** Earley’s Parser AlgorithmDECLARE ARRAY_OF_STATES Sets;function INITIALIZE(input_string)  n ← LENGTH(input_string)  Sets ← CREATE_ARRAY(n + 1)  for i ← from 0 to n    Sets[i] ← EMPTY_SET  endforfunction EARLEY_PARSER(input_string, grammar)  INITIALIZE(input_string)  n ← LENGTH(input_string)  ADD_TO_SET((*Start* → _•_S, 0), Sets[0])  for i ← from 0 to n    for each state in Sets[i]      if (state is not completed)        if (RIGHT_TO_DOT(state) is a nonterminal)          PREDICTOR(state, i, grammar)        else          SCANNER(state, i, input_string)        endif      else        COMPLETER(state, i)      endif    endfor  endfor  return Setsfunction PREDICTOR((B → *α*_•_ C*β*, j), i, grammar)  for each (C → *δ*) in GRAMMAR_RULES    ADD_STATE_TO_SET((C → _•_*δ*, i), Sets[i])   endforfunction SCANNER((B → *γ*_•_ a *δ*, j), i, input_string)  if (a is input_string[i])    ADD_STATE_TO_SET((B → *γ* a _•_
*δ*, j), Sets[i+1])  endiffunction COMPLETER((A → *δ*_•_, x), i)  for each (B (→ *γ*_•_ A *β*), j) in Sets[x]    ADD_STATE_TO_SET((B → *γ* A _•_
*β*, j), Sets[i])  endfor

## 3. Related Work

Due to the complexity of the prediction of a tertiary representation and its significant computational cost, many studies focus on predicting the earlier stage of the secondary structure. Not only is the prediction of pseudoknotted RNA structures a challenge for bioinformatics but also the annotation of pseudoknots based on the secondary or tertiary structure of RNA molecule is not an easy task [40]. Spotting pseudoknots, specifically, is known to be a challenging puzzle, considering the short experimentally verified RNA-structures-to-sequences ratio. The majority of well-known algorithms make use of dynamic programming techniques, trying to predict the lowest free-energy structure, tackling the problem in a thermodynamic approach. Typical implementations that use thermodynamic models are RNAfold [41] and manifold [42], while others such as RNAalifold [43] utilize the ViennaRNA package [41] to calculate energy minimization. Although these methods are time-consuming, requiring an exponential amount of time relative to the input sequence length; that is, the problem is NP-complete [44,45].

In this context, the bioinformatics industry adopts various approaches to overcome the efficiency barrier [46]. Stochastic methods are applied to simulate folding pathways or to sample structures [47,48,49]. An update of the specific framework utilizes the folding pathway to locate free-energy structures by determining base pairs in a deterministic way [50]. The second methodology relies on a heavily constrained dynamic programming approach. In that case, the predicted structures’ possible topologies are limited based on certain criteria [51,52,53]. Another proposed alternative is to build structures iteratively or to even utilize graph-theory techniques. Such a worth-mentioning example is the nuclear magnetic resonance (NMR)-assisted prediction of the RNA secondary-structure (NAPSS) algorithm, which includes constraints from simple NMR experiments to improve predictions [54,55,56,57]. Recently, other software tools—like RNAthor [58] and RNAProbe [59]—have appeared to facilitate the incorporation of experimental data into RNA structure prediction.

Many heuristic methods using different approaches have also been developed in the literature to overcome that computational barrier. Knotty [2] computes the secondary structure with a low runtime, using MFE prediction algorithm CCJ with sparsification. Knotty introduces a new class of structures called three-groups-of-band (TGB) and can predict a wide range of pseudoknots such as H-type pseudoknotted structures, kissing hairpins, and chains of four interleaved base pairs by overlaying TGB structures. Through the incorporation of sparsification, which improves space demands during the execution, keeping only a fraction of dynamic programming matrices, the overall need for memory remains significant. Next, ProbKnot [60] is a general secondary-structure prediction method that includes pseudoknots. It predicts base pair probabilities leveraging a partition function of any sub sequence not including pseudoknotted structures and then assembles a maximum-expected-accuracy structure from these probabilities without using dynamic programming. In that manner, it performs well in the structure prediction of pseudoknots and shows a fast execution time. A more-sophisticated variation of the latter, TheshKnot [61], outperforms its results by discarding pairs with probabilities below a given threshold. Despite that, it performs the fastest prediction and scales almost linearly to a sequence’s length, and it sacrifices accuracy compared with specialized methods for pseudoknots. In particular, IPknot [3] outperforms the systems mentioned above in terms of accuracy. It boosts the expected accuracy of a predicted structure using a thresholding variation of integer programming. It also approximates the base-pairing probabilities in order to decrease the inference time of prediction.

On the other hand, implementations with SCFGs have been proposed in the literature. These approaches present accuracy, which largely depends on the chosen SCFG that describes the secondary-structure prediction. A typical SCFGs prediction example is Pfold [62,63], which receives RNA alignment input to produce a consensus secondary structure of that as output. Additionally, a multithreaded version of Pfold, the PPfold [64], has been released. RNA-Decoder [65] predicts the secondary structure of alignments using a SCFG and also taking into account the known protein-coding context of RNAs. Various implementations exist such as Contrafold [66], Evfold [67], Infernal [68], Oxfold [69], Stemloc [70], TRNAnscan-SE [71], Xrate [72], etc., all exploiting the SCFG model. All of the above implementations are software ones, while only two implementations are hardware ones. The one in reference [73] was designed and executed on a field programmable gate array (FPGA), and the other in reference [74] was executed on a GPU using a CUDA [75] implementation of the Nussinov algorithm [76]. The two different approaches of thermodynamic models and SCFGs are more alike than different in some ways. It has been shown that Zuker’s thermodynamic model can be translated to a SCFG, by calculating the probabilities of productions from the thermodynamic constants [48]. SCFG-based as well as thermodynamic approaches aim to the optimization of an objective function; thermodynamic methods try to minimize free energy of a structure, while SCFG methods are dealing with the maximization of the corresponding probability. In these approaches, optimization lies on recursion relations and is resolved using dynamic programming techniques, leading to a computational complexity of $O(n^{3})$. Nevertheless, these approaches are diametrically opposite regarding the scientific concepts and assumptions used. Thermodynamic methods incorporate a biologically oriented, energy-driven model for RNA folding and obtain their parameters from experiments on specific short RNA molecules. SCFG-based methods, on the other hand, pursue a machine-learning orientation, by targeting on modeling the complete structures observed in nature. Afterwards, these systems reproduce similar structures, based on patterns and detected similarities. Considering that, it is obvious that SCFG-based prediction is inherently probabilistic, leveraging the advantage of probability and statistics as the background. They can also be combined with other models within the same probabilistic context to become more efficient and to improve their predictions.

Recent research also suggests the utilization of pure machine-learning approaches towards the prediction of RNA secondary structure. In [77], the authors propose using deep contextual learning for base-pair prediction, including those non-canonical and non-nested (pseudoknot) base pairs stabilized by tertiary interactions. However, the lack of sufficiently large datasets may question the quality of such deep-learning methodologies. In [78], the proposed deep-learning framework DMfold predicts the secondary structure of RNA sequences, including pseudoknots. DMfold consists of a bidirectional-LSTM network as an encoder and a fully connected layer as a decoder. The system predicts an initial dot-bracket representation for each RNA sequence given as input, using the encoder–decoder framework. Afterwards, DMfold applies the improved base pair maximization principle (IBPMP) to select the base pairs in the dot-bracket sequence and create three pseudoknot-free substructures, which in turn, are combined to calculate the secondary structures with pseudoknots. Inspired by DMfold, 2dRNA [79] proposes a coupled two-staged deep neural network, leveraging the advantages of a bidirectional LSTM with a U-net architecture. In the first stage, the two-level bidirectional LSTM encodes sequence information in higher dimensions, while a fully connected network decodes that data and predicts the dot-bracket representation. This procedure consists of the coarse-grained dot-bracket prediction (CGDBP). The second stage, called fine-grained dot-plot prediction (FGDPP), feeds that representation to a fully convolutional network, which constructs a dot-plot matrix. However, the output shows mismatches between brackets because of the inherent ability of LSTM to reveal sequential information. This problem is countered by introducing a U-net architecture, which receives that structure and predicts base-pairing, providing at the same time significant structural information. In that same context, a recent approach, ATTfold [80], predicts the secondary structure of RNA with pseudoknots. The framework utilizes deep-learning techniques based on an attention mechanism. It calculates the base-pairing score matrix via an encoder with an attention mechanism and a convolutional neural network as the decoder. Finally, the resulted matrix is enforced to comply with the hard constraints of RNA folding, and the overall architecture is trained with respect to those biological restrictions.

## 4. Overview of Our Approach—An Illustrative Example

### 4.1. The Proposed Methodology

**Figure 2:**
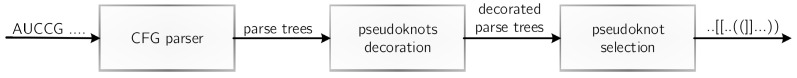
Tasks of the proposed methodology.

In the current section, an overview of the proposed methodology will be presented. The procedure of RNA pseudoknots recognition is split into the following three tasks: (i) RNA sequence is parsed using a CFG parser in order all trees that include a pseudoknot to be produced; (ii) all derived trees are then traversed to identify additional base pairs around the pseudoknot; and (iii) the optimal tree is selected via the criteria of minimum energy and the maximum number of base pairs of the pseudoknot. These three main tasks of the proposed methodology (see Figure 2) are thoroughly described in Section 4.1.1, Section 4.1.2 and Section 4.1.3, respectively.

**Figure 3:**
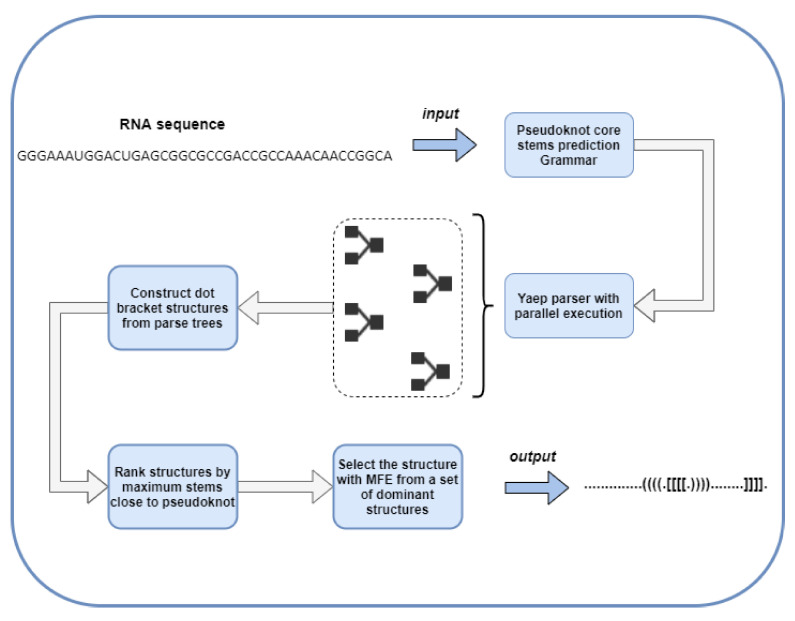
A more extensive representation of our approach.

As exhibited in Figure 2, the presented implementation given an input RNA in the form of a string representing a sequence of nitrogenous bases produces the base pairing of the given string in extended dot-bracket notation. A separate software module was developed to implement each task, and all the implementation details are described in Section 5. A more-extensive representation of our approach is shown in Figure 3.

#### 4.1.1. CFG to Identify Pseudoknots

The proposed methodology of detecting pseudoknots in sequences of nitrogenous bases representing RNA rests on syntactic-pattern-recognition techniques and specifically on an efficient CFG parser. Consequently, it is vital to select the right primitive patterns. In the case of RNA recognition, the most-typical option is to represent the nitrogenous bases adenine, cytosine, guanine, and uracil as single characters “A”, “C”, “G”, and “U”, respectively. These characters in sequence constitute an RNA representation. Hence, in our case, where a grammar parser is proposed to recognize pseudoknots in RNA, the proposed grammar vocabulary contains only the four terminal symbols T = {“A”, “C”, “G”, “U”}, with each one representing a distinct base: adenine, cytosine, guanine, and uracil, respectively. Therefore, every RNA sequence may linguistically be represented as a string containing the terminal symbols, e.g., UAGGC or AUGGCCGUACG.

The task to syntactically recognize a given pattern may actually be converted into using an appropriate pattern grammar, in order to parse the linguistic representation of the original patterns. The design of the pattern grammar may have a significant impact on the recognition’s result. Therefore, the formation of the CFG to be used is an important subtask in implementation having as the underlying model syntactic-pattern-recognition techniques. Hence, the design of an efficient grammar is indispensable in order to describe the syntax of the pseudoknot within any arbitrary RNA sequence. It is well known that CFGs are adequate to represent structural features. The $G_{RNA}$ shown in Table 1 is utilized to recognize pseudoknots in RNA.

**Figure 4:**
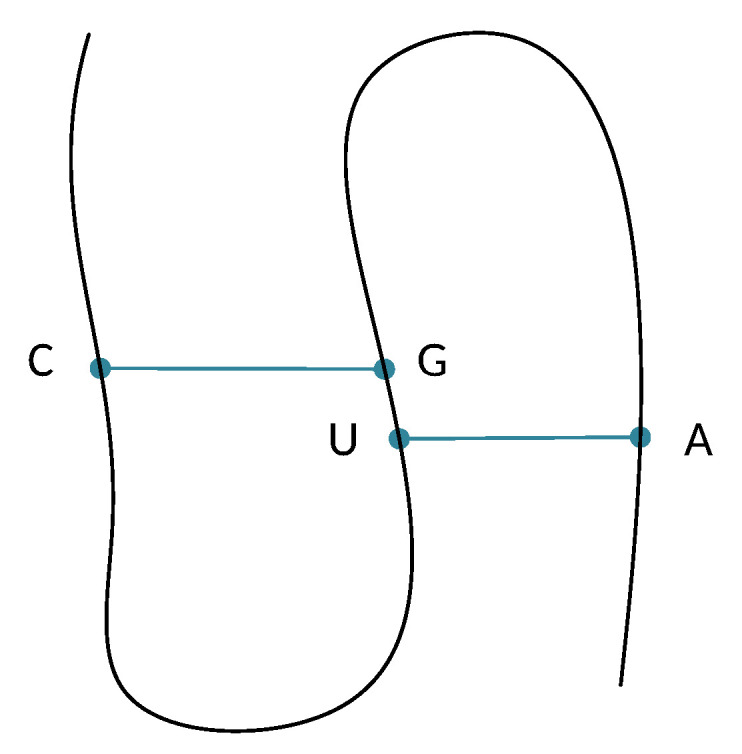
Pseudoknot detected by rule S → “C” L “U” D “G” L “A”.

The second column of Table 1 highlights all the grammar’s syntactic rules. $G_{RNA}$ contains the five non-terminal symbols of set NT = {S, L, D, K, N}. S is the start symbol; all syntactic rules having S on their left side, e.g., rule 0 to rule 15, aim to detect a possible pseudoknot in the input string. A pseudoknot consists of at least two base pairs in which half of one base pair is intercalated between the two halves of another base pair. For instance, rule 6: S → “C” L “U” D “G” L “A” specifies the existence of a pseudoknot of the form C..U..G..A where the base pairs C–G and U–A are intercalated. These base pairs for the rest of the article will be mentioned as **core stems**. Figure 4 depicts the core stems C–G and U–A of this example, while half of base pair U–A is intercalated between base pair C–G, i.e., base U is between base pair C–G. Base G belonging in base pair C–G is also intercalated between base pair U–A—that is, the paradigm of the detected interference, leading to the prediction of the pseudoknot.

L is the non-terminal symbol that will produce sequences of bases forming the two interior loops of the pseudoknot, i.e., sequences of bases between C and U as well as between G and A. Non-terminal L may produce strings belonging to set ${\left(T\right)}^{*}\neq \emptyset$, where T is the set of terminal symbols, and *∅* is the empty set. Hence, L may produce strings of length greater than zero as A, UA, CCGGAU, etc.

D is the non-terminal symbol that will produce sequences of bases between the two crossing base pairs, i.e., between bases U and G in this example. Using non-terminal symbols K and N, L may recognize substrings of terminal symbols of length zero to two, i.e., ϵ, A, U, C, G, AU, UA, AC, CA, etc. where ϵ is the empty string. The length of the sub-string between crossing base pairs may easily be extended to more than two via simple grammar modifications. The maximum length of this sub-string may be defined by the user as explained in the development environment section at knotify github repo [81].

$G_{RNA}$ may detect pseudoknots in strings where the first and last symbols of the sequence belong to the core stems group. In the examined example of a pseudoknot detected by the sixth rule, the pseudoknot exists in a substring starting from terminal symbol C and ending with terminal symbol A. However, that should not be considered a limitation since the parser is extensively executed in subparts of the strings using the sliding-windows technique.

**Figure 5:**
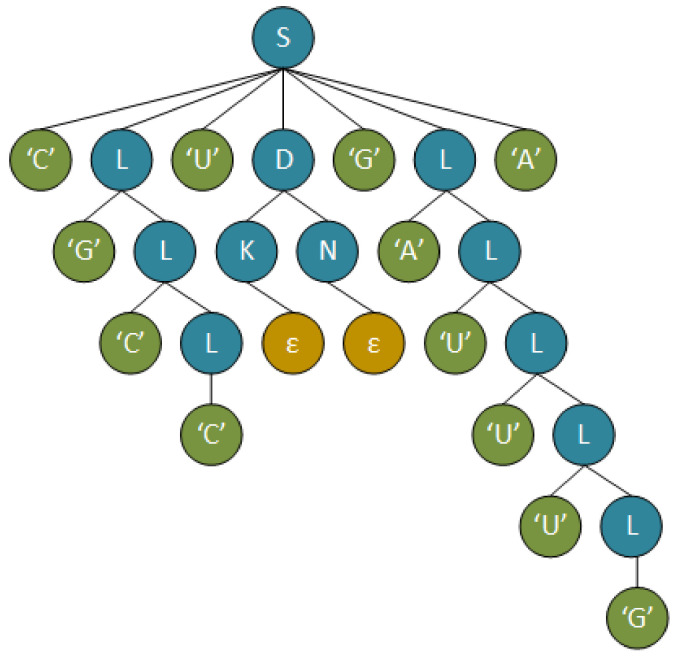
Parse-tree-recognizing pseudoknot is substring “C G C C U G A U U U G A”.

The parse tree produced by the parsing of substring “C G C C U G A U U U G A” is shown in Figure 5. Following the previous example, syntax rule 6 was used to detect the pseudoknot of the form **C** …**U G** ……**A**. Then, rules 19, 18, and 22 were used to recognize the bases between C and U, i.e., C **G C C** U G ……A. After that, rules 24, 29, and 34 were used to recognize the empty string between the U G bases of the core stems. Finally, rules 16, 17, 17, 17, and 23 were used to recognize the bases between C and U, i.e., C G C C U G **A U U U G** A. The integration of this substring in the initial RNA sequence and the process of decorating the pseudoknot with additional base pairs is explained in Section 4.1.2.

The proposed methodology parses all substrings, beginning with the one that starts with the first sequence symbol and features the minimum potential length. Iteratively, the length is extended by one symbol to include the entire initial RNA sequence finally. In the same iterative fashion, string starting points are augmented to exclude the previous set starting symbol. The parsing is over when the substring’s length to be parsed deteriorates further than a predefined threshold (i.e., the minimum length of the pseudoknot). This methodology, considering that $G_{RNA}$ is ambiguous, leads to the creation of a considerable number of parse trees. The selection methodology of the optimal tree is analyzed in Section 4.1.3. The CFG parser selected is that of YAEP [39], which is a highly efficient CFG parser based on Earley’s algorithm [33], and according to the literature, it can handle ambiguity in grammars.

Context-free grammar was selected with the view of augmenting it with attributes (forming an attribute grammar) in order to store probabilities and to manage to prune parse trees during the parse-tree construction process, in future work. In order to enhance the performance of the proposed system, an alternative implementation of the first task was proposed using a brute-force algorithm. This approach traverses the input string in order to spot all possible base pairs and then traverses all base pairs so as to identify couples of base pairs, which potentially form the core stems of a pseudoknot, i.e., consisting of two helical segments that are bound by two single-stranded sections or loops. The brute-force approach achieved a performance speed of 2.55 compared to the implementation that makes use of the context-free grammar at the first task. The speedup, though, comes at the cost of the overall extensibility that the grammar approach provides. In any case, both proposed implementations are faster than two well-known platforms [2,3]. Performance evaluation is analyzed in Section 6.

#### 4.1.2. Decorate Core Stems

Once the parse trees are generated following the methodology described in the previous subsection, all trees are traversed to decorate the pseudoknot with additional base pairs. $G_{RNA}$ is designed to identify only the pseudoknot’s core stems to enhance the CFG parser’s performance. This fact leads to a CFG with few syntactic rules and enables the parser to behave efficiently. However, the drawback is that all parse trees should be traversed to detect the rest of the base pairs surrounding and framing the pseudoknot’s core stems. All bases in each of the two pseudoknot loops are sequentially examined if they may form a base pair with another base located in a suitable position. Table 2 exhibits the part of the algorithm called **decoration**. Having detected the core stems U–A and C–G at positions 9 and 11 and 5 and 10, respectively, all bases in both pseudoknot loops, (i.e., bases at positions 6 to 9 (left loop) and 11 to 15 (right loop)) were examined to identify against forming base pairs with bases outside the pseudoknot loops. Hence, base pairs belonging in the left loop were checked out if they may form base pairs with bases at positions 17 to 19, while bases belonging in the right loop were examined to match the ones at positions 1 to 4. In both the left and the right loops of the pseudoknot, the base pairs at positions 8–17, 7–18 and 4–11, and 3–12 were sequentially detected, respectively. Table 2 presents the entire procedure in detail.

The proposed system permits the user to optionally select the existence of base pairs U–G in the pseudoknot’s loops. At the same time, the existence of bulges or interior loops in the loops of the pseudoknot is part of our future work.

#### 4.1.3. Optimal Tree Selection

According to the literature, multiple methodologies have been presented that tackle the problem of RNA base-pair prediction, with the prevalent ones being the (i) method of minimum free energy [82], which detects the RNA sequence that features the lowest amount of free energy. It is synonymous with the natural-mode structure, but it is not necessarily the structure that forms in nature. The perception of minimum free energy is basically a restatement of the second law of thermodynamics, (ii) The method of maximum pairing [83] is a technique based on the number of base pairs formed around the pseudoknot’s core stems. The dot-bracket notation with the maximum number of base pairs around the pseudoknot will probably lead to the minimum free energy. Next to the row, the (iii) method of partition function [84] is founded on the fact that the actual base pairs should entitle a high base-pairing likelihood in the estimated minimum free energy distribution. The method boosts any positive predictive value of the actual base pairs by considering their nearest neighbors’ parameters for formed free energy at a given temperature. Finally, the (iv) method of comparative sequence analysis [85] is about testing the pattern of substitutions observed in a pairwise alignment of two homologous sequences, while the (v) method of ohysical experiments [86] focuses on providing insights through actual wet experiments.

The proposed system employs a hybrid model of optimal tree selection, combining principles originating from the two most-prevalent techniques, i.e., the method of maximum pairing and that of MFE, to predict the pseudoknot pattern of any RNA secondary structure accurately yet in an efficient way. MFE is cost-effective in terms of performance; initially, all trees are sorted by the number of base pairs around the detected pseudoknot, and MFE applies only to the ones ranking the top score of the base-pairs count. This is a heuristic that outperforms the MFE original approach.

#### 4.1.4. Minimum-Free-Energy Calculation

**Figure 6:**
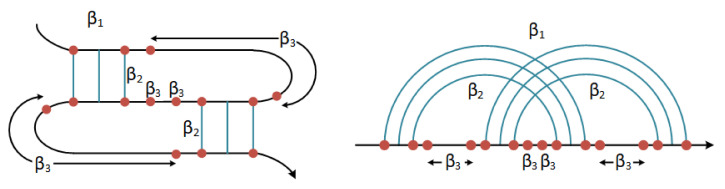
The cost of forming a pseudoknot is $\beta_{1}$, while core stems contribute a cost $\beta_{2}$ and unpaired bases inside the pseudoknot a cost $\beta_{3}$. The energies associated with the stacked base pairs were computed with respect to the standard model [87] (after [57]).

In order to select the best candidate from the set of secondary structures, our method chooses the one with the minimum free energy. To carry out this important task, a module from HotKnots [57] was incorporated, to compute the energy of each structure and then supply it to our framework for the final selection. This module is based on an algorithm introduced by Mathews [87], which has been extended for pseudoknots by Dirks [52]. Specifically, the energy of the pseudoknot is given by the relation below:

(1) $$G^{pseudo}=\beta_{1}+\beta_{2}*B^{p}+\beta_{3}*U^{p}$$

where $\beta_{1}$ is the weight for the existence of pseudoknot; $B^{p}$ is the number of core stems; and $U^{p}$ is the number of unpaired bases inside the pseudoknot. The parameters $\beta_{2}$ and $\beta_{3}$ were set to 0.1, as computed experimentally in [57], and refer to the core stems and unpaired bases, respectively. The $\beta_{1}$ weight was set to 9.6. Figure 6 provides an illustrative example for the weights-costs $\beta_{1}$, $\beta_{2}$, and $\beta_{3}$ in an indicative *H*-type pseudoknot.

## 5. Materials and Methods

### Implementation Details

According to the literature, the prediction of pseudoknots of any arbitrary RNA sequence is a NP-complete problem. On the one hand, free-energy-minimization algorithms proposed to provide pseudoknot predictions invoke dynamic programming to rank high regarding their computational cost, while their precision decreases proportionally to the length of the input sequence. On the other hand, existing heuristic approaches lack generalization capabilities when being tested under different datasets. In this setting, we introduced a novel hybrid strategy to pick the RNA sub-sequence that is the most-probable pseudoknot expression. According to Figure 3, the proposed methodology initially creates a sub-space of all potential pseudoknot expressions, i.e., successfully parsed trees that satisfy some minimum length criteria and then solves a global defined optimization problem by picking the pseudoknot representation that features (i) the maximum number of base pairs around the pseudoknot and (ii) the minimum free energy.

The proposed implementation is hybrid itself as well. By invoking Python and C code routines, a performant, scalable yet easy-to-use, and extended software package was implemented. Python was used to provide high-level flexibility and out-of-the box features such as flexible parallelization capabilities, sub-process supervision, and file management, while C nailed the parsing task by minimizing the memory footprint and maximizing CPU utilization.

**Figure 7:**
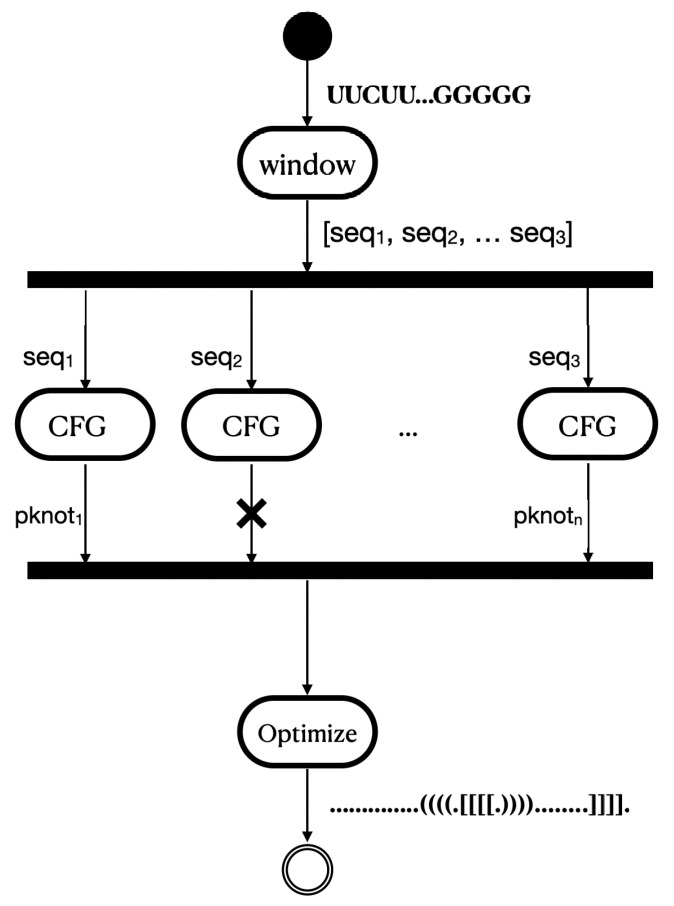
Pipeline parallelization.

The input sequence was sliced into multiple sub-sequences (this procedure is also described in Section 4.1.1). Since the tasks of parsing the derived sub-sequences are orthogonal to each other, we can parallelize the particular workload. A pool of tasks is spawned to constitute a parallel CFG parser that evaluates all the produced sub-sequences in a completely parallel fashion. The size of the pool is proportional to the CPU logical cores to max out CPU utilization, while every task is a YAEP-parser [39] instance implemented in C to guarantee optimal resource allocation and blazing-fast parsing (Figure 7).

Each CFG parser instance produces a pseudoknot structure that describes each potential pseudoknot within the CFG domain. If some parsed sub-sequences do not represent a pseudoknot, the CFG parser will fail, resulting in no pseudoknot structure. Next, all pseudoknot structures are serialized to a CSV format to be efficiently analyzed through the Pandas package [88]. Given that the resulted data frame consists of all potential solutions to our problem (i.e., valid pseudoknots), we should pick the most likely one. Assuming that the most-suitable prediction may be the one that affords the least free energy, we ended up solving a minimum-free-energy optimization problem. However, calculating the free energy for every single potential RNA folding is a computational- and memory-intensive task that sets our entire pipeline as relatively inefficient. Our methodology tackles this highly computationally intensive task by adapting the observation that free-energy minimization is directly associated with the maximization of the base-pairs count of any potential RNA folding. Consequently, instead of calculating the minimum free energy for all pseudoknot structures, a maximum stem count look-up that features O(n) time and O(n) space complexity proportional to the input sequence’s length was performed.

As mentioned in Section 2, the first task, that of pseudoknot core stems prediction, was accomplished by two different approaches: one based on the YAEP parser (knotify_yaep) and a second based on a brute-force algorithm (knotify_bruteforce). The first implementation needs O(n${}^{5}$) time: the complexity of the Earley parser [33] is for ambiguous grammars plus the complexity to traverse all direct acyclic graphs (DAG), and the YAEP parser produces as compact representation of all possible parse trees of an ambiguous grammar. On the other hand, the second one needs O(n${}^{2}$) + O(n${}^{4}$) ≈ O(n${}^{4}$): O(n${}^{2}$) to traverse the input string in order to spot all possible base pairs (the maximum number of base pairs is n${}^{2}$) and then O(n${}^{4}$) to traverse all base pairs so as to identify couples of base pairs that may form the core stems of a pseudoknot. The implementation source code is publicly available under the *knotify* github repo [81].

## 6. Performance Evaluation

### 6.1. Dataset Presentation

A dataset [1] of 262 RNA sequences was used to evaluate our methodology’s accuracy against other methodologies. It is composed of well-known RNA sequences; thus, it should be considered a perfect fit to compare our methodology against other highly respected implementations proposed in the literature, i.e., Hotknots, Iterative HFold (IHFold), IPknot, and Knotty [2,3,57,89]. The dataset of 262 RNA sequences was divided into four groups regarding their length. Consequently, there was a group of 75 RNA sequences of length smaller than 30, a group of 68 RNA sequences of length greater equal than 30 and smaller than 40, a group of 55 RNA sequences of length grater equal than 40 and smaller than 50, and a group of 64 RNA sequences of length grater equal than 50. The above-mentioned groups are notated as L < 30 (#75), 30 <= L < 40 (#68), 40 <= L < 50 (#55), and L >= 50 (#64), respectively, in the tables and figures of this section. For all methods of evaluation, the results are presented for the entire dataset and per groups.

### 6.2. Methods of Evaluation

To evaluate our methodology, we decided to go with three metrics: we asserted (i) the accuracy of the pseudoknot’s core stems prediction, (ii) the capability to predict the base pairs existing in the ground truth dot-bracket (confusion matrix), and (iii) the execution time.

#### 6.2.1. Predicting Pseudoknot location

Table 3 provides a compact comparison among our solution and the aforementioned platforms, by summarizing the capability of predicting the core stems of the pseudoknot. The comparison was made against both methodologies proposed in this article, i.e., knotify_yaep and knotify_bruteforce (see Section 4 and Section 5). Our methodology succeeded in perfectly detecting the core stems of the pseudoknot in 143 out of 262 sequences, while Knotty in 121 sequences, HotKnots in 75, IPknot in 38 sequences, and IHFold in 0 sequences. Towards the calculation of core stems’ location, we allowed one base of each pair to be located one position on the right or left, i.e., pair (*i*, *j*) is equivalent to (*i − 1*, *j*), (*i + 1*, *j*), (*i*, *j − 1*), and (*i*, *j + 1*), as proposed in [87].

The measurements divided per RNA sequences’ length are presented in Table 4, where it is shown that our methodology succeeded in predicting exactly the core stems in more pseudoknots compared to the other implementations in three out of four groups, while in the groups where the length is between 30 and 40, our methodology predicted one less than Knotty.

**Figure 8:**
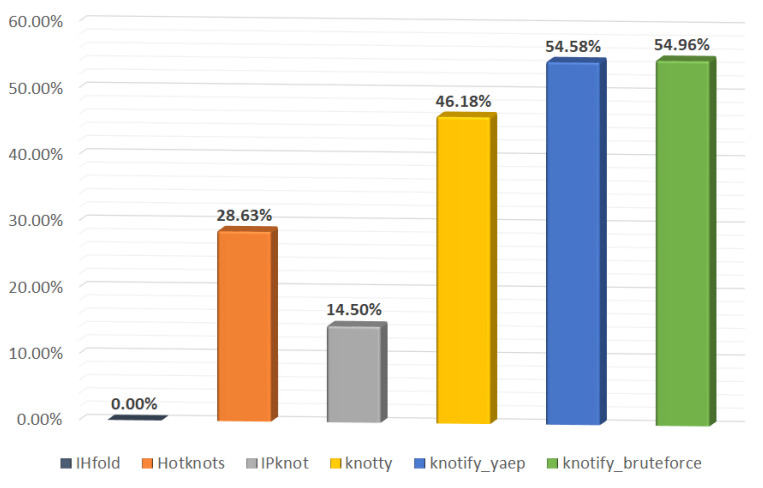
Percentage of pseudoknot’s core stems exact prediction per platform.

The percentage of pseudoknot’s core stems exact prediction per platform is also shown in Figure 8.

**Figure 9:**
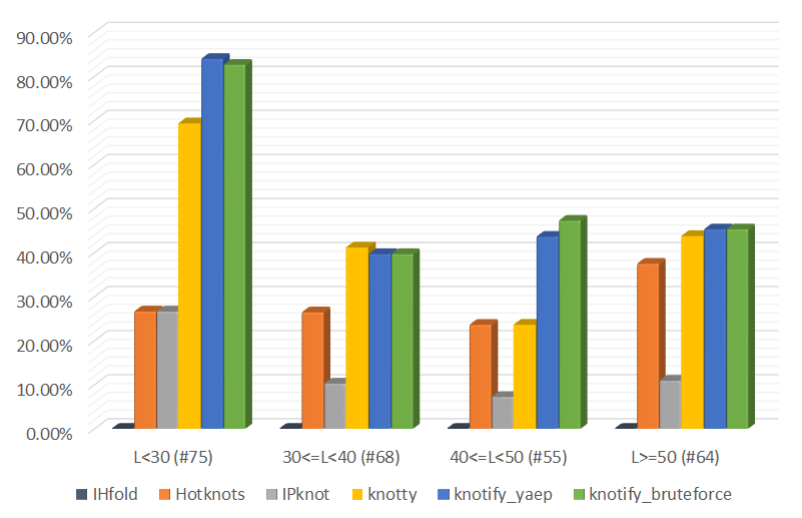
Percentage of pseudoknot’s core stems exact prediction per platform and sequence length.

The percentage of pseudoknot’s core stems exact prediction per platform for all groups of length of RNA sequences is also shown in Figure 9.

#### 6.2.2. Confusion Matrix

We benchmarked our framework on the sequences of Table 5 along with the same state-of-the-art methods IHFold, HotKnots, IPknot, and Knotty. Table 5 presents the performance for each method in terms of the positive predicted value (PPV), the recall, the F1-score, and the Matthews correlation coefficient (MCC). Equations (2)–(5) provide the definitions, where TP refers to the number of correctly predicted base pairs, FP to the number of incorrectly predicted base pairs, FN to the number of base pairs that were not predicted, and TN to the number of the bases that were not correctly predicted from the system.

(2) $$PPV=\frac{TP}{TP+FP}$$

(3) $$Recall=\frac{TP}{TP+FN}$$

(4) $$F1-Score=\frac{2\times PPV\times Recall}{PPV+Recall}$$

(5) $$MCC=\frac{TP\times TN-FP\times FN}{\sqrt{\left(TP+FP)(TP+FN)(TN+FP)(TN+FN\right)}}$$

To evaluate the overall performance, we focused on the precision, the MCC, and the F1-score. The latter is the harmonic mean of the PPV and the recall. Our methodology outperformed on average all methods in regards to precision metric having 0.784, while Knotty was 0.729, IPknot 0.718, Hotknots 0.706, and IHFold 0.608. Regardingthe F1-score and the MCC metrics, Knotty outperformed on average all methods with a F1-score equal to 0.807 and a MCC equal to 0.569. The proposed methodology had an accuracy very close to Knotty (F1-score = 0.774; MCC = 0.543). In addition, HotKnots scored F1-score = 0.738 and MCC = 0.452, while IPknot (F1-score = 0.712; MCC = 0.418) and IHFold (F1-score = 0.595; MCC = 0.226) had lower accuracy on both the F1-score and the MCC.

**Figure 10:**
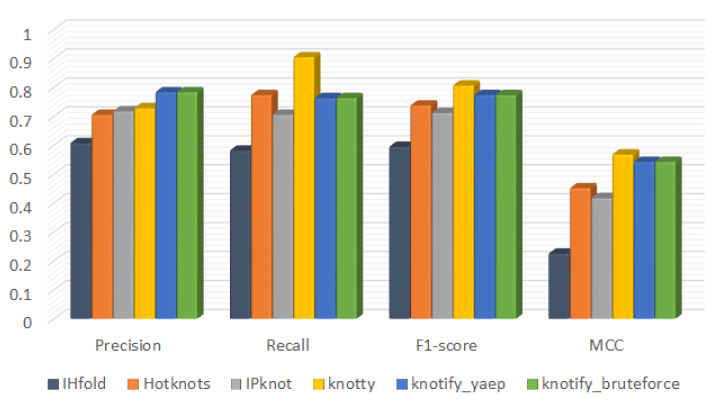
Precision, recall, F1-score, and MCC per platform.

The above-mentioned results are also shown in Figure 10.

In Table 6, Table 7, Table 8 and Table 9, the precision, the recall, the F1-score, and the MCC metrics per platform are exhibited for the four groups of different RNA sequences’ length. In these tables it is shown that our methodology outperformed on average all methods in regards to the precision metric for all ranges of length, while Knotty outperformed our methodology in regards to the F1-score and MCC metrics mainly when RNA sequences were of larger size. When the length was smaller than 30, our methodology had a higher F1-score and MCC than Knotty. As shown in Table 3, our methodology was more accurate in predicting the core stems of the pseudoknot. The increased MCC of the Knotty platform in larger RNA sequences probably correlates with the fact that larger RNA sequences incorporate multiple structures (i.e., hairpins) that do not relate explicitly to the psuedoknot one. The latter possibly augmented the overall true-positive (tp) score. One of our future work tasks is to enhance our methodology to cover even more complex patterns such as pseudoknots enclosing bulges or hairpins.

#### 6.2.3. Execution-Time Comparison

The last metric that was used in order to compare the proposed methodology with other platforms is that of execution time. In Table 10, the execution time required per platform to predict an existing pseudoknot in RNA sequences is provided. The third column of this depicts the total execution time required by each platform to analyze all 262 RNA sequences, while the second column depicts the average execution time per RNA sequence. Our methodology outperformed Knotty, which had worse results regarding the core stems prediction and the precision but better results regarding the F1-score and MCC. Knotify_bruteforce required 33.894 s; knotify_yaep required 85.756 s; and Knotty required 263.303 s. The methodology we introduced achieved a speed of 7.76 (1.004/0.129) compared to the Knoty platform. The IPknot and Hotknots performed at values of 3.45 and 1.31, respectively. Finally, IHFold recorded the lowest execution time; nonetheless, it had the poorest accuracy-evaluation profile.

**Figure 11:**
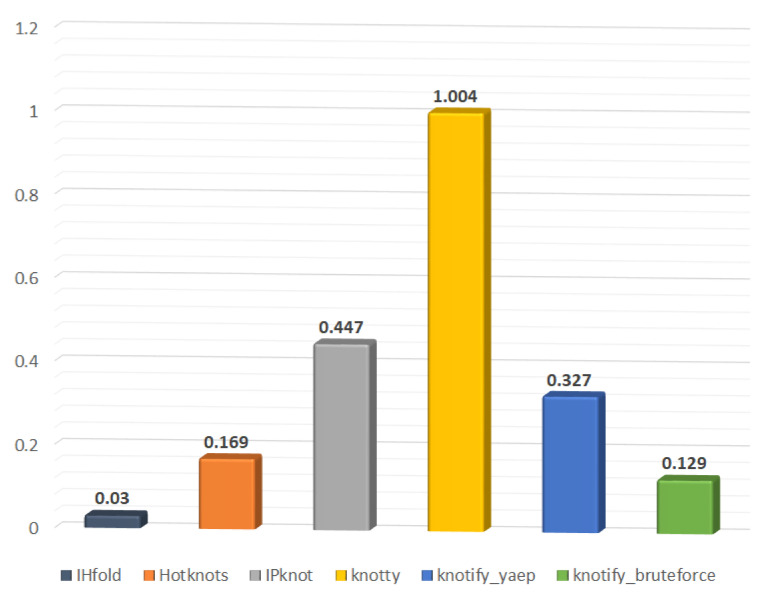
Average execution time (s) required per platform.

The execution time required per platform is also shown in Figure 11.

In Table 11, Table 12, Table 13 and Table 14, the average and total execution time per platform for the four groups of different RNA sequences’ length are shown. It is worth noting that the execution time of the Knotty platform increased significantly in proportion to the length of the input RNA sequence, while Hotknots, knotify_yaep, and knotify_bruteforce seemed to increase similarly as the length of the RNA sequence became larger, keeping a quite steady ratio.

**Figure 12:**
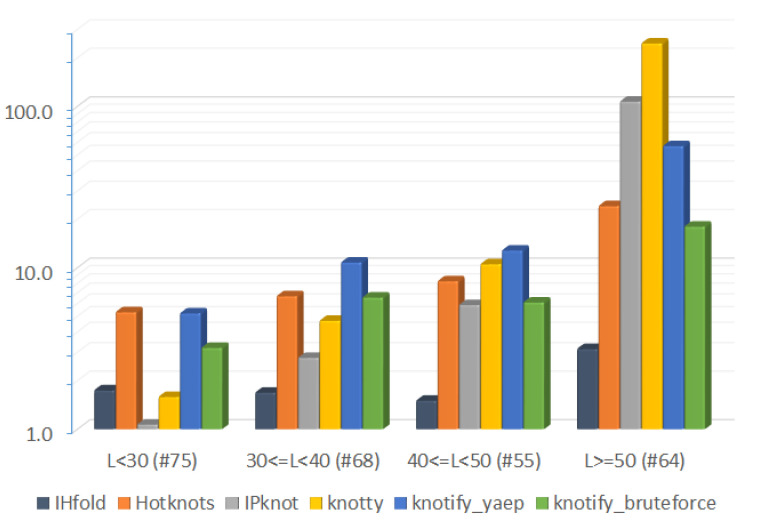
Average execution time (s) required per platform.

Total execution time required per platform for the four groups of different RNA sequences’ length is also shown in Figure 12. Please note that a logarithmic scale was used.

## 7. Discussion and Future Work

Recently, COVID-19 evolved into a severe global threat. This virus’ connection to RNA is one of the most-prominent artifacts justifying the requirement of concrete and accurate RNA-analysis toolkits. This study introduced an innovative methodology to detect H-type pseudoknots in RNA secondary structures accurately and performantly. The method is based on Early’s parser, which, given an RNA sequence in the form of a string, produces a space of all possible parse-trees, each one expressing a potential pseudoknot structure. The optimal tree is picked through a hybrid model combining pairing maximization and free-energy minimization for each context–tree pair. The evaluation of the proposed algorithm demonstrated its outperformance to the task of pseudoknot prediction, paving the way for our future work endeavours: (i) discovering even more complex patterns such as pseudoknots enclosing bulges or hairpins, (ii) building advanced searching algorithms leveraging common patterns of the secondary structure of RNA, and (iii) creating an open web platform to make our work accessible to all researchers. The work in [90,91] may be extended further to facilitate enhanced RNA-analysis services to fulfill our vision of unified collaboration among the members of an interdisciplinary team of healthcare experts since the future of medicine will be genomics.

## Figures and Tables

**Table 1:** Description of $AG_{RNA}$.

#	Syntactic Rules
0	S → “A” L “A” D “U” L “U”
1	S → “U” L “A” D “A” L “U”
2	S → “C” L “A” D “G” L “U”
3	S → “G” L “A” D “C” L “U”
4	S → “A” L “U” D “U” L “A”
5	S → “U” L “U” D “A” L “A”
6	S → “C” L “U” D “G” L “A”
7	S → “G” L “U” D “C” L “A”
8	S → “A” L “C” D “U” L “G”
9	S → “U” L “C” D “A” L “G”
10	S → “C” L “C” D “G” L “G”
11	S → “G” L “C” D “C” L “G”
12	S → “A” L “G” D “U” L “C”
13	S → “U” L “G” D “A” L “C”
14	S → “C” L “G” D “G” L “C”
15	S → “G” L “G” D “C” L “C”
16	L → “A” L
17	L → “U” L
18	L → “C” L
19	L → “G” L
20	L → “A”
21	L → “U”
22	L → “C”
23	L → “G”
24	D → K N
25	K → “A”
26	K → “U”
27	K → “C”
28	K → “G”
29	K →ϵ
30	N → “A”
31	N → “u”
32	N → “C”
33	N → “G”
34	N →ϵ

**Table 2:** Decoration of core stems of pseudoknot.

**String enumeration**	1	2	3	4	5	6	7	8	9	10	11	12	13	14	15	16	17	18	19
**String**	C	C	A	U	C	G	C	C	U	G	A	U	U	U	G	A	G	G	A
**Parser output**	.	.	.	.	[	.	.	.	(	]	.	.	.	.	.	)	.	.	.
**Step 1**	.	.	.	.	[	.	.	(	(	]	.	.	.	.	.	)	)	.	.
**Step 2**	.	.	.	.	[	.	(	(	(	]	.	.	.	.	.	)	)	)	.
**Step 3**	.	.	.	[	[	.	(	(	(	]	]	.	.	.	.	)	)	)	.
**step 4**	.	.	[	[	[	.	(	(	(	]	]	]	.	.	.	)	)	)	.

**Table 3:** Predicting pseudoknot location in entire dataset.

Platform	Exact Matches	Exact Matches (%)
IHFold	0	0
HotKnots	75	28.6
IPknot	38	14.5
Knotty	121	46.1
knotify_yaep	143	54.5
knotify_bruteforce	144	54.9

**Table 4:** Predicting pseudoknot location per RNA sequence length.

	L < 30 (#75)		30 <= L < 40 (#68)		40 <= L < 50 (#55)		L >= 50 (#64)	
**Platform**	**Exact**	**Exact**	**Exact**	**Exact**	**Exact**	**Exact**	**Exact**	**Exact**
	**Matches**	**Matches (%)**	**Matches**	**Matches (%)**	**Matches**	**Matches (%)**	**Matches**	**Matches (%)**
IHFold	0	0.00	0	0.00	0	0.00	0	0.00
Hotknots	20	26.67	18	26.47	13	23.64	24	37.5
IPknot	20	26.67	7	10.29	4	7.27	7	10.94
Knotty	52	69.33	28	41.18	13	23.64	28	43.75
knotify_yaep	63	84.00	27	39.71	24	43.64	29	45.31
knotify_bruteforce	62	82.67	27	39.71	26	47.27	29	45.31

**Table 5:** Precision, recall, F1-score, and MCC per platform in entire dataset.

Platform	tp	tn	fp	fn	Precision	Recall	F1-Score	MCC
IHFold	3056	3556	1968	2196	0.608	0.582	0.595	0.226
Hotknots	4180	3632	1744	1220	0.706	0.774	0.738	0.452
IPknot	3872	3767	1522	1615	0.718	0.706	0.712	0.418
Knotty	5026	3352	1870	528	0.729	0.905	0.807	0.569
knotify_yaep	4212	4102	1162	1300	0.784	0.764	0.774	0.543
knotify_bruteforce	4214	4101	1160	1301	0.784	0.764	0.774	0.543

**Table 6:** Precision, Recall, F1-score, and MCC per platform for sequences of length < 30.

Platform	tp	tn	fp	fn	Precision	Recall	F1-Score	MCC
IHFold	738	522	118	513	0.862	0.590	0.701	0.386
Hotknots	904	492	156	339	0.853	0.727	0.785	0.465
IPknot	916	514	124	337	0.881	0.731	0.799	0.510
Knotty	1196	469	146	80	0.891	0.937	0.914	0.722
knotify_yaep	1244	486	134	27	0.903	0.979	0.939	0.805
knotify_bruteforce	1242	485	136	28	0.901	0.978	0.938	0.802

**Table 7:** Precision, recall, F1-score, and MCC per platform for sequences of length >= 30 and < 40.

Platform	tp	tn	fp	fn	Precision	Recall	F1-Score	MCC
IHFold	550	832	352	587	0.610	0.484	0.539	0.191
Hotknots	922	851	294	254	0.758	0.784	0.771	0.528
IPknot	824	823	314	360	0.724	0.696	0.710	0.420
Knotty	1078	802	324	117	0.769	0.902	0.830	0.628
knotify_yaep	988	893	296	144	0.769	0.873	0.818	0.627
knotify_bruteforce	988	893	296	144	0.769	0.873	0.818	0.627

**Table 8:** Precision, recall, F1-score, and MCC per platform for sequences of length >= 40 and < 50.

Platform	tp	tn	fp	fn	Precision	Recall	F1-Score	MCC
IHFold	612	864	478	418	0.561	0.594	0.577	0.237
Hotknots	792	857	510	213	0.608	0.788	0.687	0.412
IPknot	764	911	410	287	0.651	0.727	0.687	0.414
Knotty	904	817	524	127	0.633	0.877	0.735	0.492
knotify_yaep	764	1010	298	300	0.719	0.718	0.719	0.490
knotify_bruteforce	772	1012	290	298	0.727	0.721	0.724	0.499

**Table 9:** Precision, recall, F1-score, and MCC per platform for sequences of length >= 50.

Platform	tp	tn	fp	fn	Precision	Recall	F1-Score	MCC
IHFold	1156	1338	1020	678	0.531	0.63	0.577	0.196
Hotknots	1562	1432	784	414	0.666	0.790	0.723	0.439
IPknot	1368	1519	674	631	0.670	0.684	0.677	0.377
Knotty	1848	1264	876	204	0.678	0.901	0.774	0.515
knotify_yaep	1216	1713	434	829	0.737	0.595	0.658	0.402
knotify_bruteforce	1212	1711	438	831	0.735	0.593	0.656	0.398

**Table 10:** Execution time required per platform in entire dataset.

Platform	Average Time (s)	Total Time (s)
IHFold	0.030	8.096
Hotknots	0.169	44.432
IPknot	0.447	117.246
Knotty	1.004	263.303
knotify_yaep	0.327	85.756
knotify_bruteforce	0.129	33.894

**Table 11:** Execution time required per platform in for RNA sequences of length < 30.

Platform	Average Time (s)	Total Time (s)
IHFold	0.0233	1.748
Hotknots	0.0709	5.314
IPknot	0.0143	1.070
Knotty	0.0212	1.590
knotify_yaep	0.0697	5.226
knotify_bruteforce	0.0427	3.204

**Table 12:** Execution time required per platform in for RNA sequences of length >= 30 and < 40.

Platform	Average Time (s)	Total Time (s)
IHFold	0.0248	1.689
Hotknots	0.0982	6.680
IPknot	0.0408	2.777
Knotty	0.0692	4.703
knotify_yaep	0.1589	10.808
knotify_bruteforce	0.0964	6.555

**Table 13:** Execution time required per platform in for RNA sequences of length >= 40 and < 50.

Platform	Average Time (s)	Total Time (s)
IHFold	0.0274	1.507
Hotknots	0.1503	8.264
IPknot	0.107	5.886
Knotty	0.1918	10.546
knotify_yaep	0.2331	12.821
knotify_bruteforce	0.111	6.103

**Table 14:** Execution time required per platform in for RNA sequences of length > 50.

Platform	Average Time (s)	Total Time (s)
IHFold	0.0492	3.151
Hotknots	0.3777	24.172
IPknot	1.679	107.511
Knotty	3.851	246.462
knotify_yaep	0.8891	56.900
knotify_bruteforce	0.2817	18.030

## Data Availability

Not applicable.

## Abbreviations

The following abbreviations are used in this manuscript:

CFG	Context-free grammar
CGDBP	Coarse-grained dot-bracket prediction
CPU	Central processing unit
CSV	Comma-separated values
CUDA	Compute unified device architecture
CYK	Cocke–Younger–Kasami
DAG	Direct acyclic graph
DNA	Deoxyribonucleic acid
FGDPP	Fine-grained dot-plot prediction
FPGA	Field programmable gate array
GPU	Graphics processing unit
IBPMP	Improved base-pair maximization principle
LSTM	Long short-term memory
MCC	Matthews correlation coefficient
MFE	Minimum free energy
NAPSS	Nuclear-magnetic-resonance-assisted prediction of secondary structure
ncRNA	Non-coding ribonucleic acid
NMR	Nuclear magnetic resonance
RNA	Ribonucleic acid
SCFG	Stochastic context-free grammar
TGB	Three-groups-of-band
YAEP	Yet another early parser
